# Translation, cross-cultural adaptation and applicability of the Brazilian version of the Frontotemporal Dementia Rating Scale (FTD-FRS)

**DOI:** 10.1590/S1980-57642013DN74000006

**Published:** 2013

**Authors:** Thais Bento Lima-Silva, Valéria Santoro Bahia, Viviane Amaral Carvalho, Henrique Cerqueira Guimarães, Paulo Caramelli, Márcio Balthazar, Benito Damasceno, Cássio Machado de Campos Bottino, Sônia Maria Dozzi Brucki, Eneida Mioshi, Ricardo Nitrini, Mônica Sanches Yassuda

**Affiliations:** 1 Neurology Department, University of São Paulo, São Paulo SP, Brazil.; 2 Behavioral and Cognitive Neurology Unit, Department of Internal Medicine, Federal University of Minas Gerais, Belo Horizonte MG, Brazil.; 3 Neuropsychology and Dementia Unit, Department of Neurology, University of Campinas, São Paulo SP, Brazil.; 4 Old Age Research Group (PROTER), Institute of Psychiatry, University of São Paulo, São Paulo SP, Brazil.; 5 Neuroscience Research Austrália, Sydney, NSW, Australia.

**Keywords:** frontotemporal lobar degeneration, behavioral variant frontotemporal dementia, Alzheimer dementia, clinical staging, disease progression

## Abstract

**BACKGROUND:**

Staging scales for dementia have been devised for grading Alzheimer's disease (AD) but do not include the specific symptoms of frontotemporal lobar degeneration (FTLD).

**OBJECTIVE:**

To translate and adapt the Frontotemporal Dementia Rating Scale (FTD-FRS) to Brazilian Portuguese.

**METHODS:**

The cross-cultural adaptation process consisted of the following steps: translation, back-translation (prepared by independent translators), discussion with specialists, and development of a final version after minor adjustments. A pilot application was carried out with 12 patients diagnosed with bvFTD and 11 with AD, matched for disease severity (CDR=1.0). The evaluation protocol included: Addenbrooke's Cognitive Examination-Revised (ACE-R), Mini-Mental State Examination (MMSE), Executive Interview (EXIT-25), Neuropsychiatric Inventory (NPI), Frontotemporal Dementia Rating Scale (FTD-FRS) and Clinical Dementia Rating scale (CDR).

**RESULTS:**

The Brazilian version of the FTD-FRS seemed appropriate for use in this country. Preliminary results revealed greater levels of disability in bvFTD than in AD patients (bvFTD: 25% mild, 50% moderate and 25% severe; AD: 36.36% mild, 63.64% moderate). It appears that the CDR underrates disease severity in bvFTD since a relevant proportion of patients rated as having mild dementia (CDR=1.0) in fact had moderate or severe levels of disability according to the FTD-FRS.

**CONCLUSION:**

The Brazilian version of the FTD-FRS seems suitable to aid staging and determining disease progression.

## Abstract (pt)

**INTRODUÇÃO:**

As escalas de estadiamento das demências, como a Clinical Dementia Rating (CDR), foram elaboradas para graduar a doença de Alzheimer (DA) e não incluem os sintomas específicos da degeneração lobar frontotemporal (DLFT).

**OBJETIVO:**

Realizar a tradução e adaptação cultural da Frontotemporal Dementia Rating Scale (FTD-FRS) para o contexto brasileiro e apresentar dados preliminares da sua aplicabilidade.

**MÉTODOS:**

O processo de adaptação transcultural consistiu em: tradução, retrotradução (realizadas por tradutores independentes), discussão com especialistas sobre a versão em português e equivalência com a versão original, desenvolvimento da versão final com pequenos ajustes. Foi feita uma aplicação piloto em 12 pacientes com diagnóstico de demência frontotemporal variante comportamental (DFTvc) e 11 com DA, pareados quanto à gravidade da demência (CDR=1). O protocolo de avaliação incluiu a Addenbrooke's Cognitive Examination-Revised (ACE-R), Mini Exame do Estado Mental (MEEM), Executive Interview (EXIT-25), Inventário Neuropsiquiátrico (INP) e a Escala de Avaliação Clínica da Demência (CDR).

**RESULTADOS:**

A FTD-FRS na versão brasileira pareceu apropriada. Resultados preliminares revelaram maiores níveis de incapacidade na DFTvc do que em pacientes com DA (DFTvc: 25% leve, 50% moderado, 25% grave; AD: 36.36% leve, 63.64% moderado). A CDR parece subestimar a gravidade da demência na DFTvc, uma vez que uma relevante proporção dos pacientes classificados com leves (CDR=1) de fato apresentaram nível moderado ou grave de comprometimento na FTD-FRS.

**CONCLUSÃO:**

A versão brasileira da FTD-FRS pode se mostrar adequada para auxiliar no estadiamento e determinar a progressão da DLFT.

## INTRODUCTION

The term Frontotemporal Lobar Degeneration (FTLD) was first introduced in 1998 by a group of Swedish and English researchers,^1^ who used it to describe a clinical syndrome characterized by progressive behavioral changes associated with atrophy of the frontal lobes and of the anterior portions of the temporal lobes. The term was introduced in order to replace terminology such as "frontal lobe degeneration of non-Alzheimer type" and "dementia of frontal lobe type".^1^ Three main conditions are described in the FTLD group: frontotemporal dementia (FTD) or behavioral variant frontotemporal dementia (bvFTD),^2,3^ semantic dementia (SD),^4^ and progressive non-fluent aphasia (PNFA).^4-6^

Recent studies have suggested that FTLD-related diseases have a significant impact on the ability to carry out daily activities. However, studies on disability severity in these conditions are scarce. In addition, disease staging in FTLD remains a challenge as most dementia staging tools have been developed for Alzheimer's disease (AD). For instance, the Clinical Dementia Rating,^7^ and other similar instruments may not capture the functional changes that are specific to FTLD. A recently developed scale specifically designed to examine the behavioral and functional changes associated with FTLD, the Frontotemporal Dementia Rating Scale (FTD-FRS), has been found to be helpful for assessing severity and the rate of functional decline.^8^

In the validation study of the FTD-FRS,^8^ by cross-sectional analyses involving a sample with three FTLD variants (bvFTD, n=29; SD, n=20; PNFA, n=28), the authors were able to identify six levels of disease severity (very mild, mild, moderate, severe, very severe and advanced/profound) with the use of the FTD-FRS. There was greater severity of functional impairment in bvFTD than in language variants, and limited correlation with cognitive measures. Follow-up analyses of a sub-sample carried out using the FRS after 12 months revealed that patients with bvFTD advanced more rapidly through the severity stages than the other variants. Therefore, the FTD-FRS was able to distinguish the functional profile of FTLD variants and identify differential rates of decline.

In Brazil, no studies investigating FTLD staging have yet been conducted and validated tools for this purpose are lacking. Therefore, the primary aim of the present study was to translate the FTD-FRS to Brazilian Portuguese and adapt it to the Brazilian cultural context.

## METHODS

The translation and cross-cultural adaptation processes consisted of the following steps: translation, back-translation (prepared by independent translators), evaluation of the back-translated version against the original version, discussion of the Portuguese version of the FTD-FRS with specialists, development of a final version after minor adjustments, and pilot application in patients with diagnoses of bvFTD and AD. The original instrument, translation, back-translation and the final version of the FTD-FRS are given in Table 1 and Appendix A. Table 2 shows percentage scores and logarithmic score conversion for the FTD-FRS correction.

**Table 1:** Original version, translation, back-translation and the final version of the FTD-FRS in Portuguese.

Question	Original Version	Translation	Backtranslation	Final Version
IntroduçãoIntroduction	For each sentence, circle the frequency of the problem on the right handside. If the question does not apply for them, e.g. he/she did not cook before, then mark N/A. Please refer to scoring and interview guides before administering the scale	À direita de cada frase, faça um círculo na frequência com que o problema ocorre. Caso a questão não se aplique, por exemplo, se a pessoa não cozinhava antes, marque como não se aplica (N/A). Por favor, consulte o manual de pontuação e aplicação da ent­revista antes de aplicar a escala	To the right of each sentence, circle the fre­quency with which the problem occurs. If the question is not applicable, for example, the person did not cook previously, mark as not applicable (N/A). Please consult the manual for scoring and application of the interview before applying the scale	À direita de cada frase, faça um círculo nafrequência com que o problema ocorre.Caso a questão não se aplique (por exemplo,se a pessoa não cozinhava antes), marquecomo "não se aplica" (N/A). Por favor, con­sulte o manual de pontuação e aplicação daentrevista antes de aplicar a escala
	**Behaviour**	**Comportamento**	**Behavior**	**Comportamento**
1	Lacks interest in doing things - their own in­terests/leisure activities/new things	Não tem interesse / se interessa por fazer as coisas - seus próprios interesses / ativi­dades de lazer / novidades	Has no interest in doing things - their own interests / leisure activities / new things	Não tem interesse em fazer as coisas - seuspróprios interesses / atividades de lazer /novidades
2	Lacks normal affection, lacks interest in fam­ily members worries	Parece distante emocionalmente, não se in­teressa por preocupações de familiares	Shows no affection, not concerned with wor­ries of family members	Parece distante emocionalmente, não se in­teressa por preocupações de familiares
3	Is uncooperative when asked to do some­thing; refuses help	Não coopera quando lhe pedem para fazer algo; recusa ajuda	Does not cooperate when asked to do some­thing; refuses help	Não coopera quando lhe pedem para fazeralgo;recusa ajuda
4	Becomes confused or muddled in unusual surroundings	Fica confuso ou desnorteado em ambientes estranhos	Becomes confused or disoriented in unfamil­iar environments	Fica confuso ou desnorteado em ambientesestranhos
5	Is restless	É agitado/inquieto	Becomes agitated/restless	É agitado/inquieto
6	Acts impulsively without thinking, lacks judgement	Age impulsivamente sem refletir, não tem bom senso	Acts impulsively without reflecting, has no discernment	Age impulsivamente sem refletir, não tembom senso
7	Forgets what day it is	Esquece em que dia está	Forgets what day it is	Esquece em que dia está
	**Outing and Shopping**	**Passeios e compras**	**Journeys and shopping**	**Passeios e compras**
8	Has problems taking his/her usual transpor­tation safely(car if has a driver licence; bike or public transport if does not have a driver licence)	Tem dificuldades para usar seu meio de transporte habitual com segurança (carro, caso tenha habilitação; bicicleta ou trans­porte público, caso não tenha habilitação)	Has problems using their usual mode of transport safely (car, if holding driving li­cense; bicycle or public transport, if not holding driving license)	Tem dificuldades para usar seu meio detransporte habitual com segurança (carro,caso tenha carteira de habilitação; bicicletaou transporte público, caso não tenha habili­tação)
9	Has difficulties shopping on their own (e.g. to go to the local shops to get milk and bread if did not use to do the main shopping)	Tem dificuldades para fazer compras sozinho (por exemplo, ir à padaria para comprar leite e pão, caso não faça as compras da casa)	Has difficulties doing shopping alone (for ex­ample, going to local shops to buy milk and bread if not doing the house shopping)	Tem dificuldades para fazer comprassozinho(por exemplo, ir à padaria para com­prar leite e pão caso não faça as comprasda casa)
	Householdchores and telephone	Tarefas domésticas e telefone	Domestictasks and telephone	Tarefas domésticas e telefone
10	Lacks interest or motivation to perform household chores that he/she used to per­form in the past	Não tem interesse ou motivação para desempenhar tarefas domésticas que realizava no passado	Has no interest or motivation to perform do­mestic tasks which they used to do in the past	Não tem interesse ou motivação para des­empenhar tarefas domésticas que realizavano passado
11	Has difficulties completing household chores adequately that he/she used to perform in the past (to the same level)	Tem dificuldade para concluir adequada­mente tarefas domésticas que realizava no passado (com a mesma qualidade)	Has difficulties completing domestic tasks properly which they used to do in the past (with the same quality)	Tem dificuldades para concluir adequada­mente tarefas domésticas que realizava nopassado (com a mesma qualidade)
12	Has difficulty finding and dialing a telephone number correctly	Tem dificuldade para encontrar e discar um número de telefone corretamente	Has difficulties finding and dialing a tele­phone number correctly	Tem dificuldade para encontrar e discar umnúmero de telefone corretamente
	**Finances**	**Finanças**	**Finances**	**Finanças**
13	Lacks interest in his/her personal affairs such as finances	Não tem interesse por seus assuntos pes­soais, como, por exemplo, suas finanças	Has no interest in their personal affairs, such as finances for example	Não tem interesse por assuntos pessoais,como, por exemplo, suas finanças
14	Has problems organising his/her finances and to pay bills (cheques, bankbook, bills)	Tem problemas para organizar suas finan­ças e pagar contas (cheques, controlar a conta do banco, contas a pagar)	Has problems organizing their finances and paying bills (cheques, managing bank ac­count, bills payable)	Tem problemas para organizar suas finan­ças e pagar contas (cheques, controlar aconta do banco e as contas a pagar)
15	Has difficulties organising his/her correspon­dence without help (writing skills)	Tem dificuldade na organização da correspondência (separar as contas, de propagan­das ou os destinatários)	Has difficulties organizing correspondence without help (writing ability)	Tem dificuldade na organização da corre­spondência (separar as contas, de propa­gandas ou os destinatários).
16	Has problems handling adequately cash in shops, petrol stations, etc (give and check change)	Tem problemas para lidar adequadamente com dinheiro em lojas, postos de gasolina, etc. (pagar e conferir o troco)	Has problems handling money properly in shops, garages, etc. (paying and checking change)	Tem problemas para lidar adequadamentecom dinheiro em lojas, postos de gasolina,etc. (pagar e conferir o troco)
	**Medications**	**Medicações**	**Medications**	**Medicações**
17	Has problems taking his/her medications at the correct time (forgets or refuses to take them)	Tem problemas para tomar suas medica­ções no horário correto (esquece ou se re­cusa a tomá-las)	Has problems taking their medications at the right time (forgets or refuses to take them) (esquece ou se recusa a tomá-las)	Tem problemas para tomar suas medica­ções no horário correto (esquece ou se re­cusa a tomá-las)
18	Has difficulties taking his/her medications as prescribed (according to the right dosage)	Tem dificuldade para tomar suas medica­ções como foram prescritas (na dosagem correta)	Has difficulties taking their medications in the manner prescribed (at the right dose)	Tem dificuldade para tomar suas medica­ções como foram prescritas (na dosagemcorreta)
	**Meal Preparation and Eating**	**Preparo de refeições e alimentação**	**Preparing meals and feeding**	**Preparo de refeições e alimentação**
19	Lacks previous interest or motivation to prepare a meal (or breakfast, sandwich) for himself/herself (rating based pre-morbid functioning; score same task for questions 19, 20 and 21))	Não tem o interesse ou motivação de costume para preparar uma refeição (ou café-da-manhã, sanduíche) para si próprio (avaliação com base no desempenho pré-morbido; pontuar a mesma tarefa para questões 19, 20 e 21)	Does not have the customary/usual interest or motivation to prepare a meal (or breakfast, snack, or sandwich) for themselves (rating based on pre-morbid performance; score the same task for questions 19, 20 and 21)	Não tem o interesse ou a motivação de cos­tume para preparar uma refeição (ou café-da-manhã, um lanche, ou sanduíche) para sipróprio (avaliação com base no desempenhopré-morbido; pontuar a mesma tarefa paraquestões 19, 20 e 21))
20	Has difficulties organizing the preparation of meals (or a snack if patient was not the maincook) (choosing ingredients; cookware; se­quence of steps)	Tem dificuldade para organizar o preparo de refeições (ou um lanche, caso o paciente não seja o responsável pela cozinha) (escolha de ingredientes; apetrechos de cozinha; sequência de passos; no preparo)	Has difficulties organizing the preparation of meals (or a snack if the patient is not respon­sible for the cooking) (choosing ingredients; cooking utensils; order of steps)	Tem dificuldade para organizar o preparode refeições (ou um lanche, caso o pacientenão seja o responsável pela cozinha) (escolha de ingredientes; apetrechos de cozinha;no preparo)
21	Has problems preparing or cooking a meal (or snack if applicable) on their own (needs supervision/help in kitchen)	Tem problemas para preparar uma refeição (ou lanche quando aplicável) sem ajuda (pre­cisa de supervisão/ajuda na cozinha)	Has problems preparing a meal (or snack when applicable) without help (needs super­vision/help in the kitchen)	Tem problemas para preparar uma refeição(ou lanche quando aplicável) sem ajuda (pre­cisa de supervisão/ajuda na cozinha)
22	Lacks initiative to eat (if not offered food, might spend the day without eating anything at all)	Não tem iniciativa para se alimentar (se não lhe oferecerem comida, pode passar o dia todo sem comer)	Has no initiative for feeding (if not offered food, can go the whole day without eating)	Não tem iniciativa para se alimentar (se nãolhe oferecerem comida, pode passar o diatodo sem comer)
23	Has difficulties choosing appropriate utensils and seasonings when eating	Tem dificuldade para selecionar os talheres e temperos apropriados quando se alimenta	Has difficulty selecting the appropriate uten­sils and condiments when feeding	Tem dificuldade para selecionar os talherese temperos apropriados quando se alimenta
24	Has problems eating meals at a normal pace and with appropriate manners	Tem problemas para comer suas refeições em um ritmo normal e de forma educada (com modos apropriados)	Has problems eating their meals at a normal pace and in an educated way (with appropri­ate manners)	Tem problemas para comer suas refeiçõesem um ritmo normal e de forma educada(com modos apropriados)
25	Wants to eat the same foods repeatedly	Quer comer as mesmas comidas repetida­mente	Wants to eat the same foods repeatedly	Quer comer as mesmas comidas repetida­mente
26	Prefers sweet foods more than before	Prefere alimentos doces, mais do que antes	Has a greater preference for sweet foods than before	Prefere alimentos doces mais do que antes
	**Self care and mobility**	**Autocuidado e mobilidade**	**Self-care and mobility**	**Autocuidado e mobilidade**
27	Has problems choosing appropriate clothing (with regard to the occasion, the weather or colour combination)	Tem problemas para escolher a vestimenta adequada (de acordo com a ocasião, o cli­ma, ou a combinação de cores)	Has problems choosing suitable attire (fitting for the occasion, weather or colour combi­nation)	Tem problemas para escolher a vestimentaadequada (de acordo com a ocasião, o cli­ma, ou a combinação de cores)
28	Isincontinent	Tem incontinência	Has incontinence	Tem incontinência
29	Cannot be left at home by himself/herself for a whole day (for safety reasons)	Não pode ser deixado sozinho em casa por um dia inteiro (por razões de segurança)	Cannot be left alone at home for a whole day (for safety reasons)	Não pode ser deixado sozinho em casa porum dia inteiro (por razões de segurança)
30	Is restricted to the bed	Está restrito à cama	Is bedridden	Está restrito à cama

**Table 2:** Percentage score and logarithmic score conversion of FTP-FRS.

Percentage score	Logit score	Category	Percentage score	Logit score	Category	Percentage score	Logit score	Category	Percentage score	Logit score	Category
100	5.39	Very mild	70	1.26	Moderate	40	-0.40	Severe	10	-3.09	Very severe
99	4.12	Very mild	69	1.07	Moderate	39	-0.59	Severe	9	-3.80	Very severe
98	4.12	Very mild	68	1.07	Moderate	38	-0.59	Severe	8	-3.80	Very severe
97	4.12	Very mild	67	1.07	Moderate	37	-0.59	Severe	7	-3.80	Very severe
96	3.35	Mild	66	0.88	Moderate	36	-0.80	Severe	6	-3.80	Very severe
95	3.35	Mild	65	0.88	Moderate	35	-0.80	Severe	5	-4.99	Very severe
94	3.35	Mild	64	0.88	Moderate	34	-0.80	Severe	4	-4.99	Very severe
93	3.35	Mild	63	0.88	Moderate	33	-0.80	Severe	3	-4.99	Very severe
92	2.86	Mild	62	0.70	Moderate	32	-1.03	Severe	2	-6.66	Profound
91	2.86	Mild	61	0.70	Moderate	31	-1.03	Severe	1	-6.66	Profound
90	2.86	Mild	60	0.70	Moderate	30	-1.03	Severe	0	-6.66	Profound
89	2.49	Mild	59	0.52	Moderate	29	-1.27	Severe	For FRS scoring: All the time = 0 Sometimes – 0 Never = 1 First. make sure that all not applicable (N/A) questions are excluded from the final score. E.g. if the patient does not take any medication then maximum score is 28 (not 30). Divide the number of “never” questions by the number of maximum applicabe questions. This percentage score should be checked against this table so that a logit score and a severity category are revealed.		
88	2.49	Mild	58	0.52	Moderate	28	-1.27	Severe			
87	2.49	Mild	57	0.52	Moderate	27	-1.27	Severe			
86	2.19	Mild	56	0.34	Moderate	26	-1.54	Severe			
85	2.19	Mild	55	0.34	Moderate	25	-1.54	Severe			
84	2.19	Mild	54	0.34	Moderate	24	-1.54	Severe			
83	2.19	Mild	53	0.34	Moderate	23	-1.54	Severe			
82	1.92	Mild	52	0.16	Moderate	22	-1.84	Severe			
81	1.92	Mild	51	0.16	Moderate	21	-1.84	Severe			
80	1.92	Mild	50	0.16	Moderate	20	-1.84	Severe			
79	1.68	Moderate	49	-0.02	Moderate	19	-2.18	Severe			
78	1.68	Moderate	48	-0.02	Moderate	18	-2.18	Severe			
77	1.68	Moderate	47	-0.02	Moderate	17	-2.18	Severe			
76	1.47	Moderate	46	-0.20	Moderate	16	-2.58	Severe			
75	1.47	Moderate	45	-0.20	Moderate	15	-2.58	Severe			
74	1.47	Moderate	44	-0.20	Moderate	14	-2.58	Severe			
73	1.47	Moderate	43	-0.20	Moderate	13	-2.58	Severe			
72	1.26	Moderate	42	-0.40	Moderate	12	-3.09	Very severe			
71	1.26	Moderate	41	-0.40	Moderate	11	-3.09	Very severe			

**Participants.** For this stage of the study it was decided to include in the research sample only patients with bvFTD. Additionally, this variant of FTLD presents features discussed in the scale (disorders of behavior and impact on activities of daily living) that could help in the detection of its applicability in Brazil.

The study sample consisted of 23 individuals aged 45 or older, with at least two years of formal education - 12 had been diagnosed with bvFTD and 11 with AD. Patients were matched for disease severity (CDR=1.0). This study was conducted from February 2011 to July in 2013.

Dementia was diagnosed according to the Diagnostic and Statistical Manual of Mental Disorders – DSMIV criteria.^9^ For the bvFTD diagnosis, the international consensus criteria were used.^2^ AD diagnosis followed the National Institute of Neurological and Communicative Disorders and Stroke and the Alzheimer's Disease and Related Disorders Association – NINCDS-ADRDA criteria for probable AD dementia.^10^

The exclusion criteria were as follows: CDR>1, visual, hearing or motor impairments which could hinder comprehension of instructions and execution of cognitive tasks, uncontrolled clinical conditions, severe psychiatric disorders, and significant cerebrovascular disease on neuroimaging.

**Evaluation procedures.** The evaluation protocol included: sociodemographic and clinical questionnaires; Addenbrooke's Cognitive Examination-Revised (ACE-R) Mini-Mental State Examination (MMSE); Executive Interview (EXIT-25).The protocol for caregivers included the Cornell Scale for Depression in Dementia, Disability Assessment for Dementia (DAD), Neuropsychiatric Inventory (NPI), the Frontotemporal Dementia Rating Scale (FRS) and Clinical Dementia Rating scale (CDR).

The ACE-R and the EXIT-25 were applied to assess cognitive performance. The ACE-R consists of a brief cognitive assessment battery testing five different cognitive domains. The highest score is 100 points, distributed as follows: attention and orientation (18); memory (35); verbal fluency (14); language (28); and visuo-spatial abilities (5). Higher scores indicate better performance. The scores regarding each of the six domains can be computed separately and their sum generates the total ACE-R score of which 30 points corresponds to the MMSE.^11,12^

The EXIT-25 assesses different aspects of executive function. It consists of 25 sub-items with scores ranging from 0 to 2, with total score ranging from 0 to 50, and lower scores indicating better performance. It assesses verbal fluency, design fluency, anomalous sentence repetition, and interference, among others. Studies have suggested that a score higher than 15 is consistent with dementia.^13,14^

For dementia staging, the CDR was completed. It evaluates six domains related to cognitive and functional performance: memory, orientation, judgment and problem solving, community affairs, home and hobbies, and personal care.^7,15^ A pre-defined algorithm allows the calculation of a total score, with 0 indicating preserved performance and higher scores indicating increased impairment.^7^

The Neuropsychiatric Inventory (NPI) in its short version is a 10-item questionnaire that makes it possible to determine the presence of neuropsychiatric and behavioral symptoms, their frequency and severity. Scores range from 0 to 144. Each behavior has a maximum score of 12 points, calculated by multiplying symptom frequency by its severity. The assessed behaviors are: delusions, hallucinations, agitation and aggression, dysphoria, anxiety, euphoria, apathy, disinhibition, irritability/lability, aberrant motor activity, nighttime behaviors, and changes in appetite. The higher the score, the greater the severity and frequency of these behaviors.^18,19^

The FTD-FRS was developed based on questions from the Cambridge Behavioral Inventory (CBI)^20^ and the Disability Assessment for Dementia (DAD).^21^ It is a 30-item questionnaire that assesses: Behavior, Outing and Shopping, Household Chores, Telephone, Finances and Correspondence, Medications, Meal Preparation, Eating, Self-care and Mobility. It was developed with the purpose of assessing disease severity and progression in FTLD.^8^ The response options for each question are: all the time=0; sometimes=0 and never =1. The examiner must add the number of alternatives marked as "never" and then divide by the number of questions answered. This will generate a percentage (an index of functional preservation) which takes into account the pre-morbid state of the patient (as the tasks which were never performed are not considered in the score). After calculating the percentage of preservation the score should be converted to a logarithm (Table 2) and the severity of the disease is established (very mild, mild, moderate, severe, very severe and profound).

The administration of the patient protocol took about 60 minutes. The interview with informants lasted about 45 minutes. The present study was approved by the Research Ethics Committee of the Hospital of Clinics, School of Medicine, University of São Paulo, under protocol number 311,601. Caregivers of patients with dementia filled out the informed consent form and were instructed regarding the research procedures.

**Statistical analysis.** The Chi-square test was used to compare categorical variables between the diagnostic groups. The Kolmogorov-Smirnov test determined the presence of a normal distribution in most of the continuous variables and therefore parametric tests were required, such as Student's *t*-test. The data were entered in the Epidata software v.3.1. For statistical analysis, the SPSS v.17.0 and the Statistica v. 7.0 software packages were used. Statistical significance was set as a p-value<0.05.

## RESULTS

**Figure 1:**
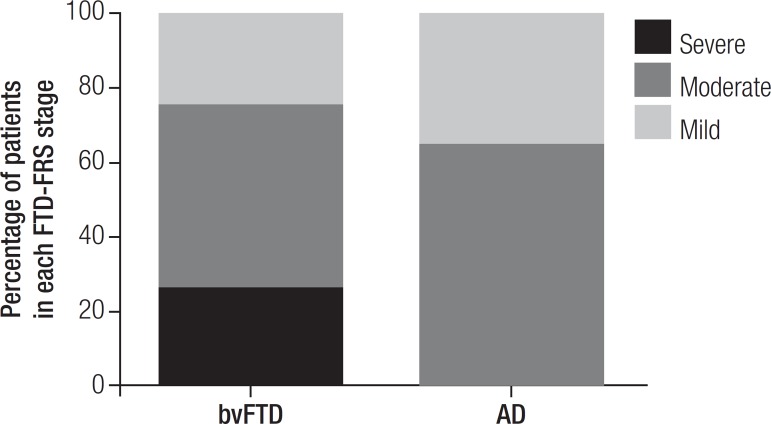
Proportion of patients in each severity category for behavioral variant frontotemporal dementia (bvFTD) and Alzheimer Disease (AD) according to Frontotemporal Dementia Rating Scale (FTD-FRS).

Table 3 shows the sociodemographic characteristics of participants. It can be noted that the groups were homogeneous with regards to gender, age and education. On the MMSE and the EXIT-25 there was a significant difference among the three groups, with the AD group exhibiting worst performance. Preliminary results for the FTD-FRS revealed greater levels of disability in bvFTD than in AD patients (bvFTD: 25% mild, 50% moderate and 25% severe; AD: 36.36% mild, 63.64% moderate), in spite of having similar CDR ratings (see Table 3 and Figure 1).

**Table 3:** Sociodemographic characteristics, cognitive performance, neuropsychiatric symptoms and severity levels for dementia sub-types.

		bvFTD (n=12)			AD (n=11)		p-value
		Means	±SD		Means	±SD	
Women (%)		33.33%			54.54%		0.305*
Age (51 to 79 years)		66.17	8.08		67.73	8.08	0.648
Schooling (4 - 20 years)		10.58	6.29		9.64	5.48	0.705
MMSE (15 to 25 points)		21.08	2.39		18.36	1.96	0.007
EXIT-25 (10 to 25 points)		18.67	3.65		15.00	3.033	0.017
ACE-R (51 to 78 points)		62.83	9.42		58.00	5.60	0.154
NPI Total (9 to 44 points)		18.83	11.15		17.00	4.92	0.621
FTD-FRS (20 to 87 points)		55.56	21.57		75.76	7.76	0.011
FTD-FRS Categories	Mild	25%			36.36%		
	Moderate	50%			63.64%		
	Severe	25%			0%		0.204*

p-value refers to Student's t-test,

* Chi-square test. 2. ACE-R: Addenbrooke's Cognitive Examination - Revised; MMSE: Mini-Mental State Examination; EXIT-25: Executive Interview; DAD: Disability Assessment for Dementia; NPI: Neuropsychiatric Inventory; FTD-FRS: Frontotemporal Dementia Rating Scale. Variations in amplitude of test scores shown in parentheses.

## DISCUSSION

In this report, we present a culturally adapted, translated version of the FTD-FRS in Brazilian Portuguese. Confrontation between original and back-translated scales, and the preliminary staging results achieved in bvFTD patients suggest that our version is suitable for clinical purposes.

Results from the scale's pilot application are in line with those from the validation study,^8^ as FTD-FRS seemed to be capable of capturing functional and behavioral change not identified by the CDR. All participants had a score on the CDR=1, and yet, according to the FTD-FRS, 25% of bvFTD patients were severely impaired. Also, in agreement with previous studies,^20,21^ our findings suggest that bvFTD is associated with greater functional loss and behavioral change compared to AD.

Determining disease severity in dementia, and especially in less prevalent sub-types, remains a controversial issue. There is currently a lack of consensus regarding the definition of severity in dementia and its ideal staging tools.^8,15,22^ Our study suggested that severity in bvFTD needs to be measured with a tool specifically designed to detect its early symptoms. Cognitive-based staging strategies are limited, since they are heavily dependent on language skills, which might overestimate disease severity, as observed in primary progressive aphasias.^23^ Additionally, in developing countries, cut-off scores in cognitive tests are unsuitable for dementia staging because of great variability in educational background. The FTD-FRS may provide a better understanding of disease progression in FTD, by showing which abilities are lost early and late in the disease, as it relies on collateral information. Also, in patients with AD, the scale showed sensitivity in detecting severity of dementia, where a great proportion of patients with a low CDR 1 had in fact moderate severity on the FTD-FRS (64%). The Brazilian version of the FDT-FRS seems suitable to aid staging and determining disease progression.

This study had some potential limitations. The dementia groups consisted of patients currently attending our clinics, which excludes more impaired patients living in nursing homes. We were unable to include neuropathology, which is ideally needed to confirm a definitive diagnosis. Additionally, the analyses were cross-sectional, restricting some of our interpretations. As to the strengths of the study, we may cite the fact that the sample was homogeneous as only early dementia cases were included (CDR=1).

Our preliminary results suggest that the Brazilian version of the FTD-FRS is appropriate for clinical use, as it was easily understood by caregivers and family members. In addition, results are in line with previous studies using the scale, as they suggested greater functional and behavioral changes among bvFTD patients. Future studies should continue to examine the psychometric characteristics of this instrument as it may play an important role in the early diagnosis of FTLD.

## APPENDIX A.

**Table:** Escala de Estadiamento e Progressão da Demência Frontotemporal Frontotemporal Dementia Rating Scale - FTD-FRS

Nome do paciente: _______________________________________________________________________________________ Data:____/____/____Respondente: ______________________________________________________________________________________________________________Relacionamento/parentesco com o paciente: ______________________________________________________________________________________				
À direita de cada frase, faça um círculo na frequência com que o problema ocorre. Caso a afirmação não se aplique, por exemplo, se a pessoa não cozinhavaantes, marque como não aplicável (N/A). Favor consultar o manual de pontuação e o roteiro de entrevistas antes de aplicar a escala (podem ser obtidos comos autores do artigo).				
**Comportamento**	**Frequência**			
1. Não tem interesse / se interessa por fazer as coisas - seus próprios interesses / atividades de lazer / novidades.	Sempre	Às vezes	Nunca	
2. Parece distante emocionalmente, não se interessa por preocupações de familiares.	Sempre	Às vezes	Nunca	
3. Não coopera quando lhe pedem para fazer algo; recusa ajuda.	Sempre	Às vezes	Nunca	
4. Fica confuso ou desnorteado em ambientes estranhos.	Sempre	Às vezes	Nunca	
5. É agitado/inquieto.	Sempre	Às vezes	Nunca	
6. Age impulsivamente sem refletir, não tem bom senso.	Sempre	Às vezes	Nunca	
7. Esquece em que dia está.	Sempre	Às vezes	Nunca	
**Passeios e compras**				
8. Tem dificuldades para usar seu meio de transporte habitual com segurança (carro, caso tenha habilitação; bicicleta ou transporte público, caso não tenha habilitação).	Sempre	Às vezes	Nunca	
9. Tem dificuldades para fazer compras sozinho (por exemplo, ir à padaria para comprar leite e pão, caso não faça as compras da casa).	Sempre	Às vezes	Nunca	N/A
**Tarefas domésticas e telefone**				
10. Não tem interesse ou motivação para desempenhar tarefas domésticas que realizava no passado.	Sempre	Às vezes	Nunca	N/A
11. Tem dificuldade para concluir adequadamente tarefas domésticas que realizava no passado (com a mesma qualidade).	Sempre	Às vezes	Nunca	N/A
12. Tem dificuldade para encontrar e discar um número de telefone corretamente.	Sempre	Às vezes	Nunca	
**Finanças**				
13. Não tem interesse por seus assuntos pessoais, como, por exemplo, suas finanças.	Sempre	Às vezes	Nunca	N/A
14. Tem problemas para organizar suas finanças e pagar contas (cheques, controlar a conta do banco, contas a pagar).	Sempre	Às vezes	Nunca	N/A
15. Tem dificuldade na organização da correspondência (separar as contas, de propagandas ou os destinatários).	Sempre	Às vezes	Nunca	N/A
16. Tem problemas para lidar adequadamente com dinheiro em lojas, postos de gasolina, etc. (pagar e conferir o troco)	Sempre	Às vezes	Nunca	
**Medicações**				
17. Tem problemas para tomar suas medicações no horário correto (esquece ou se recusa a tomá-las).	Sempre	Às vezes	Nunca	N/A
18. Tem dificuldade para tomar suas medicações como foram prescritas (na dosagem correta).	Sempre	Às vezes	Nunca	N/A
**Preparo de refeições e alimentação**				
19. Não tem o interesse ou motivação de costume para preparar uma refeição (ou café-da-manhã, sanduíche) para si próprio (avaliação com base no desempenho pré-morbido; pontuar a mesma tarefa para questões 19, 20 e 21).	Sempre	Às vezes	Nunca	N/A
20. Tem dificuldade para organizar o preparo de refeições (ou um lanche, caso o paciente não seja o responsável pela cozinha) (escolha de ingredientes; apetrechos de cozinha; sequência de passos; no preparo).	Sempre	Às vezes	Nunca	N/A
21. Tem problemas para preparar uma refeição (ou lanche quando aplicável) sem ajuda (precisa de supervisão/ajuda na cozinha).	Sempre	Às vezes	Nunca	N/A
22. Não tem iniciativa para se alimentar (se não lhe oferecerem comida, pode passar o dia todo sem comer).	Sempre	Às vezes	Nunca	
23. Tem dificuldade para selecionar os talheres e temperos apropriados quando se alimenta.	Sempre	Às vezes	Nunca	
24. Tem problemas para comer suas refeições em um ritmo normal e de forma educada (com modos apropriados).	Sempre	Às vezes	Nunca	
25. Quer comer as mesmas comidas repetidamente.	Sempre	Às vezes	Nunca	
26. Prefere alimentos doces, mais do que antes.	Sempre	Às vezes	Nunca	
**Autocuidado e mobilidade**				
27. Tem problemas para escolher a vestimenta adequada (de acordo com a ocasião, o clima, ou a combinação de cores).	Sempre	Às vezes	Nunca	
28. Tem incontinência.	Sempre	Às vezes	Nunca	
29. Não pode ser deixado sozinho em casa por um dia inteiro (por razões de segurança).	Sempre	Às vezes	Nunca	
30. Está restrito à cama.	Sempre	Às vezes	Nunca	
**Outras observações:**				
